# 3D Printed Beam with Optimized Internal Structure—Experimental and Numerical Approach

**DOI:** 10.3390/ma18245512

**Published:** 2025-12-08

**Authors:** David Juracka, Petr Lehner, Marek Kawulok, David Bujdos, Martin Krejsa

**Affiliations:** 1 Department of Structural Mechanics, Faculty of Civil Engineering, VSB—Technical University of Ostrava, L. Podéště 1875, 70800 Ostrava, Czech Republic; 2 Department of Building Materials and Diagnostics of Structures, Faculty of Civil Engineering, VSB—Technical University of Ostrava, L. Podéště 1875, 70800 Ostrava, Czech Republic

**Keywords:** 3D printing, algorithm, structure, polycarbonate, four-point bending

## Abstract

This article compares the results of numerical and experimental analysis of the mechanical properties of an optimized 3D-printed beam. The samples were subjected to a four-point bending test, and corresponding numerical models were created at the same time. The beams were printed using 3D printing and their weight was reduced by using an internal spatial grid with variable thickness that gradually increases towards the outer walls. This approach allows for effective optimization of material strength while minimizing raw material consumption during production. One of the key findings is the determination of the ultimate strength between fibers, the mode of failure, and the high agreement between the experimental results and the numerical model using the finite element method. The optimized beam achieved nearly 60% weight reduction while maintaining comparable load-bearing capacity. The knowledge gained opens up new possibilities in the field of materials engineering and also makes a significant contribution to the methodology of developing and optimizing these structures using 3D printing technology.

## 1. Introduction

In the contemporary landscape, the increasing dependence on the potentials of roboticization and process automation is evident. This domain encompasses additive manufacturing, notably 3D printing, which has experienced significant growth in the past decade, impacting nearly every industry. The capacity to produce objects based on created or generated models has found practical applications across diverse sectors, including design, construction, engineering, space exploration, medicine, and fashion. This versatile technology allows for the working with various materials such as plastic, metal, ceramics, and even tissues, supported by a myriad of printers prioritizing efficiency based on factors such as speed, strength, cost, and weight [1]. The cross-section of the element cannot be considered homogeneous, since there are gaps between the individual fibers. For this reason, the elements have an anisotropic behavior and therefore it is important to specify whether the stress is conducted along the fibers, across, or at a certain angle [2,3,4,5,6]. The mechanical properties of these elements are further influenced by the material used, the temperature of the nozzle and printing plate, the layer thickness, the fiber width, and the fiber adhesion. Furthermore, it is necessary to consider the internal filling of the object formed by a geometric pattern and the percentage filling of the defined space [7,8,9,10].

The profound interconnection of 3D printing technology with different industries underscores the far-reaching consequences of advances in printing processes and model design. Previous research mainly focused on uniform or periodic infill patterns, while our study explores a parametric algorithm generating a continuous gradient structure. The novelty lies in coupling local geometry control with variable wall thickness, which enables tailoring stiffness distribution within the beam. An intriguing application lies in the realm of creating small yet uniquely shaped bearing elements, potentially valuable for reconstruction, repairs, and civil or mechanical engineering. The design of such elements poses challenges to ensure the necessary structural properties, ease of 3D printing, and optimal utilization of material. For beam-shaped elements, the achievement of these goals can be facilitated through the intelligent definition of internal infills. An interesting use of 3D printing in construction is, for example, the study [11], where printed lost formwork is used for columns of various shapes. However, it is also possible to use the printed internal structure, which is complicated in shape, as a kind of substitute for the reinforcement in the beams [12]. In this article, the infill is taken to the next level, because until now the infill is only printed in one fiber thickness. If the fill is thicker, it is another complex object inside the main object and thus has its own infill. For simplicity, let us call it the internal structure. Several articles [10,11,12,13,14,15,16] have already dealt with the topic of 3D structure design and optimization.

One way of preparing numerical models of 3D printed samples is to use finite element method (FEM)-based software. In this case, 3D printing can be viewed at the microscale, where we model each individual layer of the print with gaps [13], or at the macroscale, where we consider the sample as a homogeneous whole with orthotropic material properties [14]. In this paper, the latter approach is chosen because the samples were printed with high precision and to achieve high durability and low delamination. The paper presents the experimental results of the four-point bending test in comparison with the numerical results of the FEM model. The force-deflection diagram and failure modes compared to the equivalent stress are investigated. The main goal of this study is to validate the performance of this algorithmically optimized gradient structure through both experimental four-point bending tests and corresponding finite element simulations. This study focuses on the validation of one optimized gradient pattern. Comparison of multiple topologies will be the subject of future research.

## 2. Materials and Methods

### 2.1. Algorithm for Generated Structure

Most 3D-printed objects consist of an outer cover and infill, which can vary in design, strength, and printing speed. Previous studies focused on adjusting fillers [15,16,17,18,19], but current geometry options allow limited modifications to density and direction. Traditional methods of adjusting the filling density involve manual steps. Expanding on this, conventional print preparation programs interpret each virtual closed object as necessitating a preset geometric filling. By adopting an inverse approach and creating an internal virtual model (essentially generating empty cavities), the internal filling becomes constrained by its boundaries. Consequently, a lightweight internal structure is formed with fill, transitioning from a gradient fill to a gradient structure. The algorithm, developed within the Rhino parametric program with the assistance of the Grasshopper sub-program, empowers the creation of an internal structure within any designated 3D object. A multitude of parameters influence its morphology, including density, grid size along individual axes, structure thickness, outer wall thickness, and more. The algorithm’s key strength lies in its ability to dynamically adjust the thickness of this structure, introducing a gradual and gradient transformation. These characteristics are customizable on a global and local scale, offering versatile control over the object’s internal composition. This is a key feature of similar procedures [20]. Unlike the gradient infill methods in studies [16,20], which modify density using fixed interpolation functions, the proposed approach directly varies wall thickness according to spatial stress distribution predicted by FE pre-analysis.

### 2.2. Sample Geometry and Material Properties

**Figure 1:**
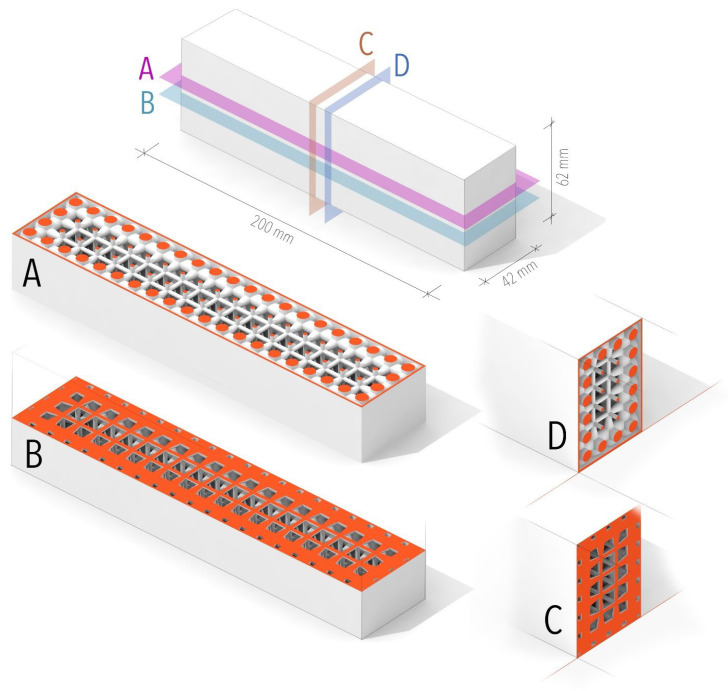
Schematic of longitudinal and transverse sections showing the different internal structures of the specimen. A, B—longitudinal horizontal sections. C, D—vertical cross sections.

For experimental analysis, 5 samples were printed and their digital twins were prepared in FEM software ANSYS 2024 R2 for numerical analysis. The sample has dimensions of 200 × 42 × 62 mm (See Figure 1). This shape was fed into a program with an algorithm that was set to create a regular internal structure along axes 10 mm apart with a minimum thickness of 2 mm and a maximum of 7.5 mm at the edges. The thickness of the outer walls of the sample is 1 mm. These parameters were chosen based on a preliminary FEM analysis and allow for reproducibility of the design. The thickness of the internal structure increases as the distance from the external walls of the object shortens, which results in a linear increase in the amount of mass towards the edges in all three axes.

The aim of printing such shapes is, of course, to obtain a lighter sample, saving material while maintaining the strength and durability of the sample. The present research mainly focuses on the comparison of experimental data and the evaluation of the numerical model, and therefore a solid sample is not included. Table 1 shows a comparison of the weight, i.e., the amount of material, between the structured and solid beam, and the printing time. By comparison, a structured sample is half as light as a solid but also requires more print time. For comparison, a solid beam model was also analyzed numerically to establish the baseline mechanical response.

One of the strongest materials for this type of printer—PC Blend (polycarbonate)—was selected for production. The samples were processed on the Prusa i3 MK3S+ 3D printer (Praha 7, Czech Republic) with PrusaSlicer preparation software [21]. For each specimen, a full straight fill with a uniform fiber direction was chosen according to the set with fiber dimensions of 0.4 mm to 0.2 mm. A file was generated from the preparatory software to instruct the 3D printer. The mechanical properties of the material according to the manufacturer [21] indicate these values (see Table 2). Each specimen was printed in a flat orientation with the main fibers aligned along the longitudinal axis. The nozzle temperature was 265 °C, bed temperature 100 °C, and layer height 0.2 mm.

## 3. Experimental and Numerical Analysis

### 3.1. Four-Point Bending Test

**Figure 2:**
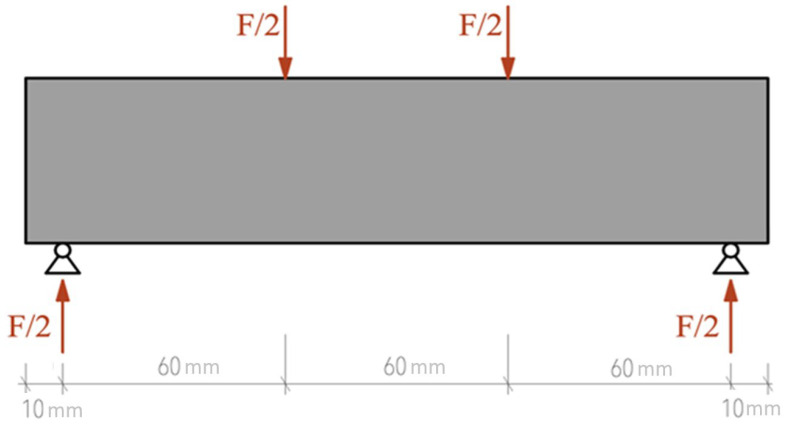
Static diagram of a four-point bending load test.

The experimental and numerical program was based on the four-point bending test (see Figure 2). Five specimens were printed for the experiments. The objectives were to obtain a force–displacement diagram, to achieve full failure of the specimens, and to obtain a record of the crack path. For these purposes, video recordings of each test were taken and data from the electromechanical testing machine were stored. A displacement sensor was placed directly under the load position.

**Figure 3:**
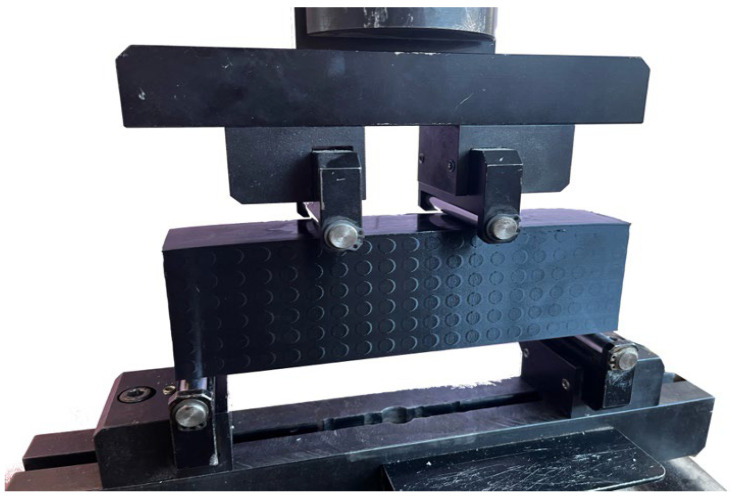
Sample prepared for four-point bending test in an electromechanical testing machine.

Unlike three-point bending, the influence of concentrated stress at the load point is minimized, allowing for better observation of the actual behavior of the material and the formation of cracks and fractures. In the case of the 3D-printed samples examined, the four-point bend allowed for detailed observation of the deformation process, the nature of the failure, and the localization of shear stresses, which became the decisive factor in sudden failure. Figure 3 shows a photograph of the sample prepared for the test. The test was carried out on a universal test machine Formtest 300 kN (Seidner & Co. GmbH, Riedlingen, Germany). The span between supports was 180 mm and the distance between the two loading points was 60 mm. A displacement transducer was placed under midspan and strain gauges were attached on the lower surface near the center.

### 3.2. Numerical Model

**Figure 4:**
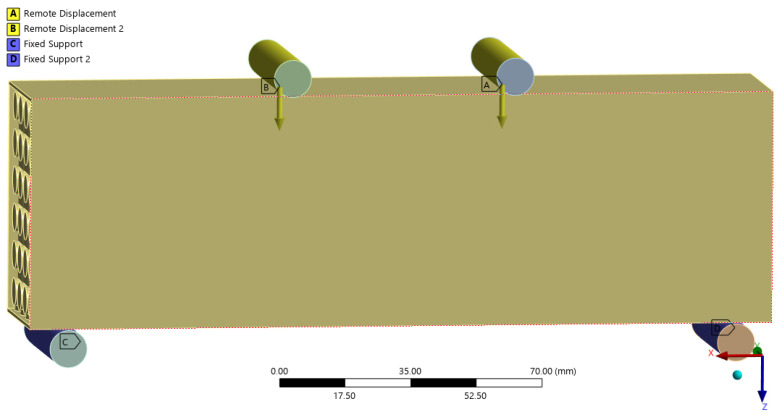
Numerical model with boundary conditions—the side is exposed to show the structure.

For the preparation and analysis, a 3D model based on the finite element method was created in ANSYS 2024 R2 software [22]. The geometry fully corresponds to reality without scale. The boundary conditions of the model are based on the real dimensions and loads, and the model also includes steel distribution rollers (see Figure 4), which have set frictional contact surfaces, the coefficient of friction was set to 0.3.

The lower spreading rollers are anchored fixed; the upper spreading rollers transmit the displacement imitating the displacement of the press head. This makes it possible to compare the force–displacement diagram between experiments and the model. The material properties were obtained from previous laboratory measurements [23] are listed in Table 2. The model contains approximately 554,000 nodes and 322,000 elements. Finite element type was SOLID186. The finite element mesh was set to an average element size of 1 mm. Further details on the numerical model of the same setup can be found in other studies [14,24]. Although the material parameters were isotropic in Table 2, the model incorporated orthotropic elasticity directions aligned with the print fibers, defined using local coordinate systems. A solid beam model without internal cavities was simulated under identical boundary conditions for comparison of stiffness and maximum stress.

## 4. Results

**Figure 5:**
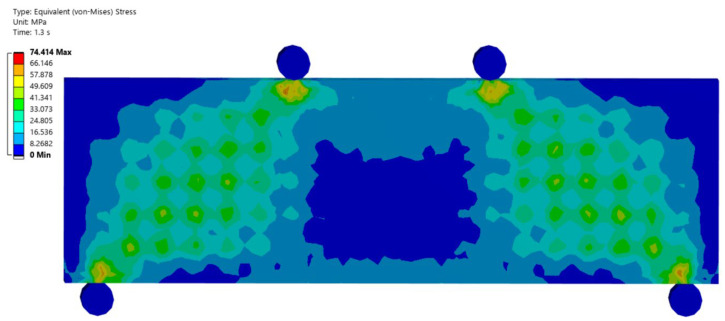
Front view of the numerical model: graphical representation of the von-Mises equivalent stress at the moment of exceeding the critical strain.

**Figure 6:**
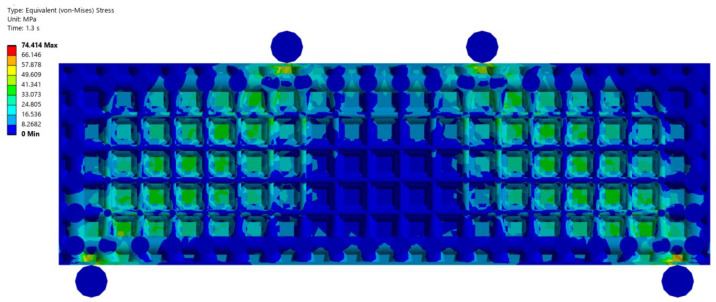
Vertical longitudinal section of the numerical model: graphical representation of the von-Mises equivalent stress at the moment of exceeding the critical strain.

The aim of the research was to analyze the similarities and differences between the experimental test and the numerical model on the four-point bending test. With the numerical model and previous experience from other material tests, it was possible to estimate the failure mode of the specimen. Figure 5 shows the equivalent stress distribution of the von-Mises numerical model from the frontal view at the critical limit of strain. Figure 6 shows the same equivalent von-Mises stress in a longitudinal vertical section of samples.

**Figure 7:**
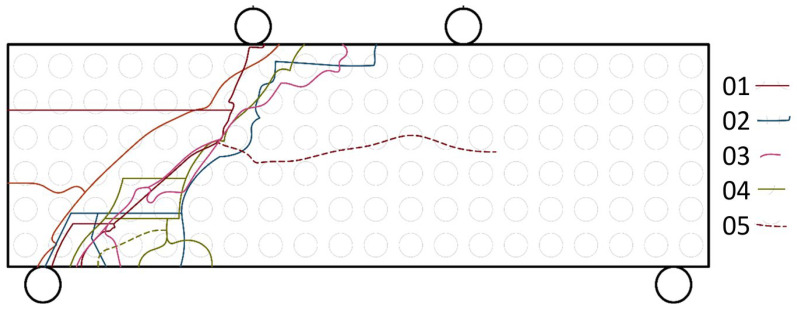
Schematic drawing of crack paths from the frontal view—any color is from different sample.

For the purpose of comparing the numerical model and the experiments, crack paths were recorded and plotted using high-speed camera analysis (see Figure 7). Considering this stress distribution, it was expected that the crack paths in the loaded specimens would be directed from the bottom supports to the top ones, as observed in the specimens.

**Figure 8:**
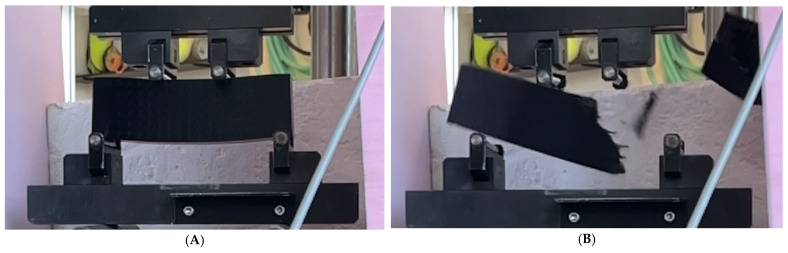
Images of sample 01 obtained from video recording: (**A**) sample just before failure, (**B**) sample just after failure.

The location and shape of these lines indicate the cause of failure, which is shear failure. Due to the speed of the fracture (even when shooting in slow motion at 240 frames per second), it is impossible to clearly determine where the origin of the fracture is located. Another detail emerging from these lines is that almost all samples always had more fragmentation on the lower side than on the upper side closer to the center where the load was applied. Figure 8 shows two images taken from a video recording during the test of sample no. 01. These images show the deflection of the sample just before failure and then the sample just after failure.

**Figure 9:**
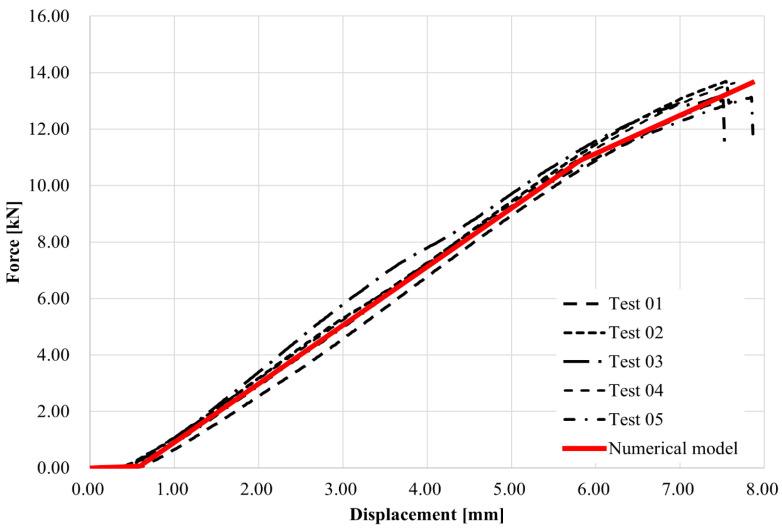
Force–displacement diagram form experimental and numerical analysis.

The main contribution of the data obtained was the possibility to compare the force–displacement diagram (see Figure 9). In the first part of the curves, close to 0.7 mm displacement, the press head seating is observed. Furthermore, the curves from the experiments and from the numerical model are at a very similar level with high agreement. Both the model and the experiments show a change in behavior at the force level of about 11 kN, which is due to the change in elongation of each part of the optimized structured shape. The total failure of the specimens occurred at a level close to 14 kN, which was also achieved by the numerical model at the same strain. It should be added to the test procedure that failure of the specimens occurred very quickly without warning. The specimens exhibited high internal stresses with no equivalent strain. The optimized beam reached 14 kN ultimate load with 267 g of material, while the solid beam model achieved 15 kN with 634 g, confirming nearly identical load capacity at less than half the weight. This demonstrates that optimization achieved 60% material reduction with minimal loss in strength.

## 5. Discussion

The results of experimental tests and numerical simulations showed very good agreement in terms of load curves and the nature of sample failure. The four-point bending test confirmed that beams with a gradient-optimized internal structure exhibit a high degree of predictability in their behavior, with the finite element-based numerical model accurately capturing both the ultimate load-bearing capacity and the deformation curve. Although the final failure occurred suddenly and without noticeable plastic deformation, the nature of the failure—mainly shear type—was the same for all samples and corresponded to the assumptions from the stress fields of the numerical model. An interesting finding is that the optimized internal filling allows for significant weight savings, while the load-bearing capacity remains comparable to samples made of solid material [16,17]. This confirms the effectiveness of the algorithmically generated internal structure, which not only reduces raw material consumption and thus production costs, but also contributes to the sustainability of 3D printing technology. Future work will include simulations of larger-span four-point bending and three-point bending to monitor the transition to tensile failure. The disadvantage is the longer printing time, which is about 150% as long as that of a solid sample. However, this factor may be acceptable in practice given the material savings achieved and the overall reduction in weight of the element. The novelty of this study lies in demonstrating that gradient internal structures, generated through a parametric algorithm rather than predefined infill templates, can be directly implemented in common 3D printing workflows. Future research will extend the approach to beams of different aspect ratios to explore tensile and shear failure transitions, as well as alternative infill topologies for further optimization. Extension to a parametric study with alternative internal structures was not possible within the scope of this project, but will be included in subsequent research.

## 6. Conclusions

The aim of the research and the paper was to investigate the properties of a specimen with an internal gradient structure under four-point bending stress. This internal structure is generated by a proprietary algorithm and aims to save material and distribute the internal stress transfer. A highly accurate numerical model incorporating accurate geometry and boundary conditions was prepared for real printed specimens.

During the experiments, the specimen failed suddenly without plastic strain, indicating failure from shear forces. All specimens failed in the same manner with very similar cracks path. The waveforms are identical from the equivalent stress distribution. Additionally, the force–displacement diagram from the experiments and from the numerical model shows high agreement. The gradient-optimized beam achieved about 60% weight reduction while maintaining 93% of the load capacity of a solid beam. The combination of numerical prediction and algorithmic control provides a promising basis for future adaptive structural design in additive manufacturing.

Thanks to the numerical model, it is possible to analyze the internal structure in more detail, on the other hand, thanks to the experimental testing, it is possible to find the exact crack paths and obtain further information. The above experience will lead to the extension of the types of internal structure in the future research in order to increase the performance towards material consumption. The novelty of this study lies in the use of a parametric algorithm for gradient structures, which allows for a 60% material saving while maintaining 93% of the load-bearing capacity. This approach has potential for lightweight structural elements and sustainable 3D printing.

## Figures and Tables

**Table 1:** Comparison of weight and printing time.

Type of Sample	Weight (g)	Printing Time (Hours)
Structured	267	33.16
Solid	634	20.16

**Table 2:** Material properties [21].

Parameter	PC Blend
Density [kg·m^3^]	1220
Modulus of elasticity [GPa]	1.90
Poisson ratio [-]	0.35
Tensile strength [MPa]	63

## Data Availability

The original contributions presented in this study are included in the article. Further inquiries can be directed to the corresponding author.
